# Dysgraphia in Patients with Primary Lateral Sclerosis: A Speech-Based Rehearsal Deficit?

**DOI:** 10.1155/2008/825042

**Published:** 2008-12-18

**Authors:** S. Zago, B. Poletti, M. Corbo, L. Adobbati, V. Silani

**Affiliations:** ^1^Department of Neurological SciencesUniversity of Milan Medical SchoolIRCCS Ospedale Maggiore-PoliclinicoMangiagalli e Regina Elena–IRCCS Istituto Auxologico ItalianoMilanoItaly; ^2^Department of Neurology and Laboratory of Neuroscience‘Dino Ferrari Center’University of Milan Medical SchoolIRCCS Istituto Auxologico ItalianoMilanoItaly

**Keywords:** Primary lateral sclerosis, speechless condition, dysgraphia, rehearsal deficit

## Abstract

The present study aims to demonstrate that errors when writing are more common than expected in patients affected by primary lateral sclerosis (PLS) with severe dysarthria or complete mutism, independent of spasticity. Sixteen patients meeting Pringle’s et al. [34] criteria for PLS underwent standard neuropsychological tasks and evaluation of writing. We assessed writing abilities in spelling through dictation in which a set of words, non-words and short phrases were presented orally and by composing words using a set of preformed letters. Finally, a written copying task was performed with the same words. Relative to controls, PLS patients made a greater number of spelling errors in all writing conditions, but not in copy task. The error types included: omissions, transpositions, insertions and letter substitutions. These were equally distributed on the writing task and the composition of words with a set of preformed letters. This pattern of performance is consistent with a spelling impairment. The results are consistent with the concept that written production is critically dependent on the subvocal articulatory mechanism of rehearsal, perhaps at the level of retaining the sequence of graphemes in a graphemic buffer. In PLS patients a disturbance in rehearsal opportunity may affect the correct sequencing/assembly of an orthographic representation in the written process.

## 1. Introduction

Investigations of writing organization within the central nervous system have considerably benefited from the analysis of dissociated deficit reported in patients with localized or widespread cerebral injuries [17]. Two primary variants of dysgraphia have been described: *central dysgraphia*, which refers to linguistic problems leading to errors in word spelling and *peripheral dysgraphia*, which reflects modality-specific distortion in written letter formation or typing [13]. In particular, *spelling* refers to the central ability to retrieve or to assemble an orthographic representation, or a code or sequence of letters [9].

Cognitive neuropsychologists apply information processing models of normal cognition to the analysis of disorders of higher cortical functions, including writing. A number of single patients have substantiated the hypothesis that the writing process involves a series of components, each dedicated to a particular aspect of the process as a whole [5,26,39]. A widely accepted general model of spelling of single words distinguishes two routes for translating between phonology and orthography. Via a *lexical process*, words could be accessed from a component devoted to the acoustic/phonological analysis of verbal information and a phonological lexicon in which phonology of familiar words is available. The information is later transferred to the semantic mediation level, and then to the orthographic lexicon in which the internal representations of words are

[1]A preliminary version of this paper was presented to the 14th International Symposium on ALS/MND, Milan, Italy 17–19 November 2003.

ISSN 0953-4180/08/$17.00 © 2008 – IOS Press and the authors. All rights reserved stored. The model also contains a *sublexical process* involved in assembling spelling for unfamiliar letter strings in which the whole stimulus could be segmented into its component phonemes and followed by applications of phoneme to grapheme transformation. According to some theorists both lexical and sub-lexical processes converge upon a common short-term mechanism termed as the 'graphemic buffer' or 'orthographic output buffer'. The characteristics of graphemic buffer disorders in writing have been described as consisting of omissions, substitutions, insertions and movement errors (or a combination of these) and include a stimulus length effect in spelling words and non-words [6]. The influence of a range of lexical variables such as word frequency, age of acquisition, imageability and neighbourhood size in spelling accuracy has already been discussed [3,37]. The graphemic buffer is engaged in order to achieve a written instance of specified internal orthographic representations in terms of graphic details or letter shapes prior to accessing the graphic motor procedure which specifies the actual strokes required to construct letters. This standard multi-component model has generally guided the burgeoning research in this area for further elaboration of the writing process.

In a preliminary report, we briefly described the dysgraphic pattern of six patients with primary lateral sclerosis (PLS), a rare motor neuron disease variant characterized by a progressive involvement of precentral pyramidal neurons with secondary pyramidal tract degeneration but lacking in lower motor neuron involvement [23,34]. We hypothesized that a dysfunction of the rehearsal mechanism, not usually considered by standard writing models, could be the cause of writing errors in PLS [41]. Although never investigated in detail, other studies suggest the theorical possibility of a writing/spelling impairment in PLS patients [24, 33]. In particular, Piquard et al. [33] clinically observed spelling errors during a dictation task and declared that: '*Grammatical errors found in writing language in PLS patients might be a consequence of a frontal lobe dysfunction. [. . .] Further studies with a more systematic analysis of written language are needed in order to verify the existence of dysorthographia in PLS and possibly in ALS*.

In this paper we present the writing analysis of a group of sixteen consecutive PLS patients presenting different levels a dysarthria leading in some cases to a condition of total loss of speech. The inability to use oral language induces some of them to use written language as their only form of communication. Our attention was attracted to the observation that the written production (spontaneous or dictated) of these patients, even if legible and informative, was often mildly agrammatic and characterized by spelling errors. At the most peripheral level the graphic or articulatory details were substantially preserved. This dysgraphic pattern was also informally confirmed by family members. The purpose of this work is to test the written performance of PLS patients and to give a possible functional and anatomical interpretation of the writing/spelling deficit.

## 2. Methods and patients

We analysed sixteen PLS patients (5 male and 11 female) with an average age of 67.56 $+/-$ 8.59 years and an educational level of 8.50 years $+/-$ 4.31. Average disease duration at examination was 4.06 years (range 3–8 years). A diagnosis of PLS was established on the basis of Pringle's neurological criteria [34]: adult onset and progressive insidious disease course $>$3 years; absence of family history; nearly symmetrical, bilateral pyramidal involvement, including the face; normal serum chemistry; CSF negative for oligoclonal bands and negative tests for syphilis, Lyme disease, human T lymphocytic virus 1 and 2; absence of denervation potentials on electromyography; absence of compressive lesions of cervical spine or foramen magnum on spinal MRI; absence of high demyelinated signal lesions outside the cortical tract through brain MRI. All patients developed a progressive dysarthria culminating in some cases in a condition of total loss of speech with variable difficulties in balance and weakness and stiffness of the legs. Spasticity was scored according to the modified *Ashworth scale*: grade 0 (no increase in tone), grade 1 (slight increase in tone, giving a 'catch' when the affected part is moved), grade 2 (more pronounced increase in tone, but affected part easily flexed), grade 3 (considerable increase in tone; passive movement difficult), grade 4 (affected part rigid in flexion or extension) [28]. Bulbar involvement was graded according to the *Amyotrophic Lateral Sclerosis Functional Rating Scale – Revised* (*ALSFRS-R*) bulbar sub-score (speech 0–4; sialorrhea 0–4; swallowing 0–4) [7]. Table 1 describes the patients sample.

All PLS cases underwent two standard neuropsychological tests to evaluate auditory understanding (*Token Test* of De Renzi and Faglioni [11]) and non-verbal intelligence (*Raven's Coloured Progressive Matrices* of Raven [36]). The average value of PLS patients at the *Token Test* was 30.75/36 (range 19–36; s.d. 4.04), while

Table 1
Clinical features of Primary Lateral Sclerois (PLS) patients

| | Patients | | | | | | | | | | | | | | | |
|---|---|---|---|---|---|---|---|---|---|---|---|---|---|---|---|---|
| | 1 | 2 | 3 | 4 | 5 | 6 | 7 | 8 | 9 | 10 | 11 | 12 | 13 | 14 | 15 | 16 |
| Sex | F | F | F | F | M | M | M | F | M | F | F | F | M | F | F | F |
| Education (yrs) | 5 | 8 | 8 | 8 | 8 | 17 | 13 | 5 | 5 | 5 | 8 | 8 | 17 | 13 | 5 | 3 |
| Age of onset | 59 | 78 | 61 | 70 | 52 | 69 | 50 | 56 | 73 | 61 | 73 | 62 | 56 | 67 | 75 | 74 |
| Family history | − | − | − | − | − | − | − | − | − | − | − | − | − | − | − | − |
| Duration of disease (yrs) | 5 | 3 | 8 | 3 | 3 | 3 | 3 | 5 | 3 | 3 | 3 | 3 | 4 | 3 | 3 | 10 |
| Site of onset | B | B | LL | LL | B | LL | B | LL | LL | LL | B | UL | LL | LL | B | LL |
| Spasticity (Ashworth) | 2 | 1 | 3 | 3 | 1 | 4 | 2 | 2 | 2 | 3 | 1 | 2 | 4 | 2 | 2 | 3 |
| Pseudobulbar affect | − | + | − | − | + | − | − | − | + | + | + | − | − | − | − | + |
| ALSFRS-R bulbar sub-score | 5 | 0 | 8 | 11 | 8 | 9 | 11 | 11 | 9 | 8 | 5 | 11 | 8 | 4 | 3 | 7 |
| Tongue denervation (EMG) | − | − | − | − | − | − | − | − | − | − | − | − | − | − | − | − |

Abbreviations: PLS, Primary Lateral Sclerosis; + = present; − = absent/negative; M, Male; F, Female; B, Bulbar; UL, Upper Limb; LL, Lower Limb; Ashworth scale 0–4; ALSFRS-R, Amyotrophic Lateral Sclerosis Functional Rating Scale – Revised bulbar sub-score 0–12; EMG, electromyography.

the average value of *Raven's test* was 27.28/36 (range 20–33, s.d. 3.49). The cut-off values of the Italian normative study were respectively >29/36 on the *Token Test* [11] and >18.6 on the *Raven*'s one [2].

We also carried out a writing examination consisting of three different tasks. In the first, the patient was asked to write a set of dictated words ($n = 15$) of different length (from 4 to 9 letters) and rank of frequency (5 low, 5 medium, 5 high) in accordance with De Mauro et al. [10], along with a set of nonsense words ($n = 10$) of different lengths (from 4 to 9 letters). In a second task the patient was instructed to compose the same words and non-words by sorting preformed letters displayed on a scrabble tile set.

The words and non-words were also presented in printed form for the copying task. Finally, five short phrases selected from the *Esame del Linguaggio* [15], were presented for dictation and copy tasks. Sixteen age, gender and educational level matched control subjects with no known neurological abnormalities also completed the task for comparison.

## 3. Results

Statistics variables were described as percentages or means ± standard deviation (SD). Difference in proportions and means were calculated using a Student's $t$ test. In addition, Pearson's correlation coefficient ($\rho$) was calculated to establish the relationships between ALSFRS-R bulbar score and dysgraphic degree (number of errors) in PLS patients. A $p$-value of less than 0.05 was considered to be statistically significant.

Of sixteen patients, 14 made two or more errors in writing tasks, while only 6 controls made two or more errors and 10 made no errors at all. The error types detected included deletions, transpositions, additions, and letter substitutions. Due to the fact that quantification and differentiation of errors presented some difficulties with certain words, because of the occurrence of mixed errors (e.g. MARINAIO (sailor) → MARIDANIO, showing here addition and transposition errors at the same time), we deemed more appropriate computing a maximum of one error for each item. Samples of some PLS errors are reported in Fig. 1 and in Table 2.

The results of the PLS patients along with control subjects, in both writing through dictation and through copying (words, non words, phrases) can be found in Table 3. It is possible to observe the high number of errors detected in PLS patients for the dictation conditions and minimal errors in the copying task when compared with normal subjects. The errors were more frequent in the writing on dictation of sentences and non-words.

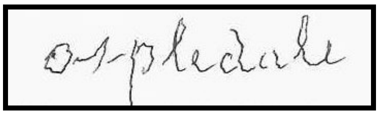

Despite the limited number of used stimuli we observed that PLS patients' performance with words was influenced by stimulus length (4–5 letters = 0.0% errors; 6–7 letters = 34.26% errors; 8-9 letters = 65.74% errors), but not by the word rank frequency (low frequency words = 35.3% errors; medium frequency words = 33.8%; high frequency words = 30.9% errors). The stimulus length effect was significantly evident also with non-words (4–5 letters = 3.9% errors; 6–7 letters = 40.74% errors; 8–9 letters = 56.17% errors). Although the most obvious interpretation of these results is that the patients have a graphemic buffer disorder, our hypothesis is that a subvocal articulatory rehearsal mechanism is responsible for (or contributes to) the spelling deficit. We informally noted that the greater number of errors was observed in patients with absence of articulatory movement and in addition, statistically, the number of error was correlated with the ospedale (hospital)

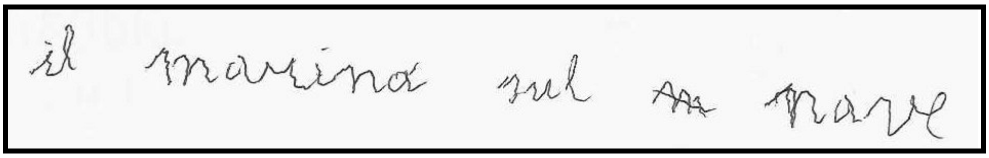

Il marinaio sale sulla nave (the sailor-man boards the boat)

Fig. 1. Example of writing errors detected in PLS patients.

Table 2
Type of errors detected in PLS patients

| Error types | | | | |
|---|---|---|---|---|
| Substitution | orologio | (*clock*) | → | orolo**c**io |
| | lunedì | (*Monday* ) | → | lun**i**di |
| | canguro | (*kangaroo*) | → | can**c**uro |
| | emiticio | (*non-word*) | → | emiti**f**io |
| | catampo | (*non-word*) | → | catam**b**o |
| Addition | ospedale | (*hospital*) | → | osp**l**edale |
| | settembre | (*september*) | → | sette**n**mbre |
| | orologio | (*clock*) | → | orolog**g**io |
| | medico | (*doctor*) | → | medi**to**co |
| Deletion | Italia | (*Italy*) | → | ital_a |
| | piastrella | (*tile*) | → | pi_strella |
| | azzurro | (*blue*) | → | az_urro |
| Shift/Deletion | settembre | (*september*) | → | se**mtt**e_re |
| | Lombardia | (*Lombardia*) | → | lom**d**__i**b**a |
| Shift/Exchange | violidia | (*non-word*) | → | vio**dil**ia |
| Addition/Deletion | inverno | (*winter*) | → | i**n**n__r**v**o |
| | lunedì | (*monday*) | → | l**i**_n_di |
| Shift/Addition | marinaio | (*sailor-man*) | → | mari**dan**io |
| Substitution/Add. | bilancia | (*balance*) | → | **m**ilan**g**ia |

dysarthric disorder degree at the ALSFRS-bulbar score (Pearson's correlation coefficient ($\rho$) 0.605, $p < 0.01$) but not with disease onset ($\rho$ 0.009, $p > 0.97$).

These data converged with the recent observation of Lucchelli and Papagno [24] that in PLS patients writing errors appear and progress at the same rate as speech difficulties.

## 4. Discussion

In the present study we have set out the results of a writing evaluation on a group of PLS' patients. Analysis revealed a disproportionate number of errors, compared to normal subjects, in the dictation writing task compared with the copying task. Errors included omissions, transpositions, insertions and letter substitutions. The errors were observable on writing through dictation and on writing with a set of preformed letters. In addition, at the most peripheral level, the graphic details were recognizable or well specified. This pattern of performance has been usually attributed to a central writing impairment.

Although the poor number of stimuli analyzed prevent a sophisticated analysis in terms of the buffer process, see [3,37], it is relevant that the writing errors appear more evident in PLS patients with an extreme degree of articulatory deficit; this suggests the possibility that an overt defective articulation could possibly impair subvocal articulation which prevents the correct sequencing/assembly of letters and syllables in writing tasks.

A legacy between defective (or abolished) articulation and a writing/spelling disorder was established in

Table 3
Characteristics and mean number of errors detected in PLS patients

| | PLS patients | Controls | Test | $p$-value |
|---|---|---|---|---|
| Mean number of errors (type of task) ± ds | | | | |
| Dictation | 6.18 ± 8.59 | 0.75 ± 1.18 | $t$-test | 0.0236 |
| words | 1.88 ± 3.28 | 0.06 ± 0.25 | $t$-test | 0.0436 |
| non-words | 2.44 ± 3.03 | 0.69 ± 1.20 | $t$-test | 0.0445 |
| sentences | 1.88 ± 3.12 | 0 | $t$-test | 0.0295 |
| Copy | 1.25 ± 2.77 | 0.38 ± 0.72 | $t$-test | n.s |
| words | 0.06 ± 0.25 | 0.13 ± 0.34 | $t$-test | n.s |
| non-words | 0.44 ± 1.21 | 0.19 ± 0.54 | $t$-test | n.s |
| sentences | 0.75 ± 1.57 | 0.06 ± 0.25 | $t$-test | n.s |
| Writing with preformed letters | 4.50 ± 5.28 | 1.38 ± 1.63 | $t$-test | 0.0364 |
| words | 2.06 ± 3.04 | 0.50 ± 1.26 | $t$-test | n.s |
| non-words | 2.44 ± 2.66 | 0.88 ± 1.31 | $t$-test | 0.0466 |
| Mean number of errors (type of stimuli) ± ds | | | | |
| Total errors | 11.94 ± 15.42 | 2.50 ± 3.14 | $t$-test | 0.0288 |
| words | 4.00 ± 5.97 | 0.69 ± 1.54 | $t$-test | 0.0462 |
| non-words | 5.31 ± 6.27 | 1.75 ± 2.49 | $t$-test | 0.0477 |
| sentences | 2.63 ± 4.43 | 0.06 ± 0.25 | $t$-test | 0.0353 |

the 1950s. Luria observed that in the initial phases of learning to write children do not do so in silence, but repeat each word loudly. At first, the repetition of words to be written is carried out in a loud, as opposed to a low voice, then reduced to a whisper and finally seeming to disappear [25]. Luria states that written language is accompained for long periods by an unexpressed 'inner speech'. Recording the minimum larynx's movements during writing, he shows that such movements also persist when writing is fully developed. Luria also reported a series of works in which patients with important impairments of the articulatory system (e.g. palatoschisi) show an increase of mistakes during writing tasks [25]. In recent years numerous studies have documented a depreciation in writing performance with spelling errors in an induced articulatory suppression condition [4,38,29,22].

In addition, few recorded clinical cases can be found in which loss of speech has been related to the writing/spelling process. For example, Ferguson and Boller [20] described three patients who displayed writing/spelling errors in combination with a motor speech disturbance and impaired motor limb function. Their conclusion was that the agraphia occurred because of the combination of disordered feedback from the motor speech apparatus and limbs. More recently, Lucchelli and Papagno [24] reported interesting observations in the study of a patient with a slow progressive neurodegenerative anarthria. A writing breakdown consisting in deletions, substitutions or transpositions with no lexical-semantic deficit or non response was documented. The authors argue that a defective overt articulation could impair a non-linguistic subvocal rehearsal module that interacts with writing performance. It was notable that in this case there was an evolution of the writing deficit parallel to articulatory deficit. Papagno and Girelli [30] reported the inability to write under articulatory suppression (repeat the syllable 'bla') in a progressively dysgraphic patient. They argued that the patient cannot write because the task precludes the rehearsal of the phonological representation of words. Also Duffy, Peach and Strand [12] informally observed mispelling errors during writing in some patients with progressive apraxia of speech and motor neuron disease.

The neural correlates of the writing deficit shown by our PLS patients remains elusive because of the inconsistency of the CT and MRI radiological information. We can only make some speculative hypothesis. We could consider the possibility that loss of speech in our PLS patients is probably linked to a degeneration of the projections that via the spinal cord are sent to the motor speech area (glosso-kinaesthetic centre) in the lower and posterior part of the (left) frontal lobe. Here the activities of the various muscles which subserve speech (labial, lingual, palatal, laryngeal and respiratory) are combined in the necessary patterns to produce articulate speech. The adjacent Broca's region (Brodman's 44/45 areas) has been related to the speech-based subvocal rehearsal process, a mechanism conceived as an active silent process that refreshes the contents of the passive phonological store, thus preventing trace decay. A small, but growing, body of active studies are in line with this hypothesis. For example Paulesu, Frith and Frackowiak [31], in a PET study with normal subjects, aiming to separate the functional anatomy of the subvocal rehearsal system from the phonological store, showed that the premotor frontal area 44 Broadmann (Broca's area) was activated during the mental rehearsal process. Awh, Smith and Jonides [1] in a PET study identified the Broca's area as playing a central role in mental rehearsal. McGuire et al. [27] in another PET study showed that inner speech (i.e. phonological representation without covert articulation) was associated with activation of the left inferior frontal gyrus (Brodmann area 44/45). Moreover they underlined that the left premotor cortex and the left posterior superior temporal gyrus were activated during an auditory verbal imagery task, but not during inner speech. Fiez et al. [21] in a PET study concluded that left frontal opercular regions appear to be involved specifically in the rehearsal of verbal material. Chein and Fiez [8] using fMRI found two left inferior regions (dorsal BA 44 and ventral BA 45) and the SMA (medial BA 6) to be active during rehearsal of verbal maintenance. Interestingly these authors predicted that regions involved in rehearsal would demonstrate sensitivity to stimulus factors, including articulatory lenght and lexicality that can influence the articulatory control process. In other words, articulatory rehearsal should not be thought of as a single component operating as a continuous loop, but that it may be further fractionated into subcomponents that show changing levels of engagement as rehearsal proceeds.

In addition to this neuroimaging evidence, some recent and less recent clinical studies have related damage of the frontal cortices with a deficit in writing/spelling. Interestingly, a number of these patients (but not all) present an articulatory deficit to varying degrees. For example Eskridge and Parkhill [16] reported a patient with agraphia associated with a cyst in Exner's area. Exner [14] in fact is usually credited with first describing agraphia in association with lesions of the superior aspects of the left premotor cortex, namely, the region above areas 44 and 45 (Broca's area) often referred to as *Exner's area*. In the Eskridge and Parkhill case, agraphia was characterised by poorly formed or omitted letters and spelling errors. The patients were not aphasic and reading ability was relatively well preserved, although it was noted that *'he not infrequently omits and misstates words'*. Penfield and Roberts [32] describe a patient who developed transient agraphia following an extensive excision of the second and third frontal convolutions below Broadmann's field 8 on the left hemisphere. The patient's agraphia which resolved within 3 months was characterised primarily by agrammatism. During the period that the patient was agraphic, he was not aphasic and reading was normal.

More recently, a patient with agraphia associated with a focal lesion of the left precental gyrus, immediately posterior to Exner's area was described by Rapsack et al. [35]. Despite the fact that the lesion was confined to the motor cortex, motor strength was normal. The patient's agraphia was characterized as a lexical agraphia, that is, letters were well formed but written spelling of irregular words was impaired relative to spelling of regular words and orthographical nonwords. The patient was not alexic but oral spelling mirrored the pattern of written spelling. Also Hillis et al. [19] studied five patients with acute left hemisphere ischemic stroke in the posterior left frontal regions (Broadmann's areas 44 and 45) with a probable impaired access to orthographic representations for written output. Hillis and colleagues hypothesized that the posterior, inferior frontal gyrus are essential for converting abstract lexical representation to modality-specific motor output and selection of morphological forms. The authors therefore suggested a pure linguistic basis of the writing ability deficit in these patients. However, only two patients in this study had an articulatory deficit, thus complicating evidence for our hypothesis [18,19]. On the other hand we are aware that not all severe dysarthric and anarthric patients show misspelling during writing [40].

In short, although we could not completely reconcile the wide literature mentioned above, it seems likely that frontal regions are recruited during writing to refresh orthographic representation and that in PLS patients limited subvocal rehearsal opportunity prevents the correct sequencing/assembly of letters and syllables in writing.

## Acknowledgement

Preparation of this article was supported by a donation of the Cattani's Family.

## References

[1] E. Awh, E.E. Smith and J. Jonides, Human rehearsal processes and the frontal lobes: PET evidence, *Annals of New York Academy of Sciences* **769** (1995), 97–117.
[2] A. Basso, E. Capitani and M. Laiacona, Raven's coloured progressive matrices: normative values on 305 adult normal controls, *Functional Neurology* **2** (1987), 189–194.
[3] A. Buchwald and B. Rapp, Rethinking the graphemic buffer?, *Brain and Language* **91** (2004), 100–101.
[4] C. Burani, G. Vallar and G. Bottini, Articulatory coding and phonological judgements on written words and pictures: The role of the phonological output buffer, *The European Journal of Cognitive Psychology* **3** (1991), 379–398.

[5] A. Caramazza, G. Miceli, G. Villa and C. Romani, The role of the graphemic buffer in spelling: evidence from a case of acquired dysgraphia, *Cognition* **26** (1987), 59–85.
[6] A. Caramazza and G. Miceli, The structure of graphemic representations, *Cognition* **37** (1990), 243–297.
[7] J.M. Cedarbaum, N. Stambler, E. Malta et al., The ALSFRS-R: a revised ALS functional rating scale that incorporates assessments of respiratory function. BDNF ALS Study Group (Phase III), *Journal of the Neurological Sciences* **169** (1999), 13–21.
[8] J.M. Chein and F.J. Feiz, Dissociation of verbal working memory system components using a delayed serial recall task, *Cerebral Cortex* **11** (2001), 1003–1014.
[9] P. De Bastiani and C. Barry, A cognitive analysis of an acquired dysgraphic patient with an 'allographic' writing disorder, *Cognitive Neuropsychology* **6** (1989), 25–41.
[10] T. De Mauro, F. Mancini, M. Vedovelli and M. Voghera, *Lessico di frequenza dell'italiano parlato*, Fondazione IBM Italia, Etaslibri, Milano, 1993.
[11] E. De Renzi and P. Faglioni, Normative data and screening power of a shortened version of the Token Test, *Cortex* **14** (1978), 41–49.
[12] J.R. Duffy, R.K. Peach and E.A. Strand, Progressive apraxia of speech as a sign of motor neuron disease, *American Journal of Speech-Language Pathology* **16** (2007), 198–208.
[13] A.W. Ellis, Spelling and writing (and reading and speaking), in: *Normality and Pathology in Cognitive Functions*, A.W. Ellis, ed., Academic Press, London, 1982.
[14] S. Exner, *Untersuchungen über die Localisation der Functionen in der Grosshirnrinde des Menschen*, W Braumuller, Vienna, 1881.
[15] *Esame del Linguaggio. Manuale*. Associazione per lo Sviluppo delle Ricerche Neuropsicologiche, Milano, Esame elaborato dal Centro di Neuropsicologia dell'Università di Milano, 1974.
[16] J.T. Eskridge and C. Parkhill, Cyst of the brain in the foot of the left second frontal convolution, *Medical News* **11** (1896), 122–129.
[17] A.E. Hillis, Progress in cognitive neuroscience research on dysgraphia: introduction, *Neurocase* **10** (2004), 89–90.
[18] A.E. Hillis, R.J. Wityk, P.B. Barker and A. Caramazza, Neural regions essential for writing verbs, *Nature Neuroscience* **6** (2003), 19–20.
[19] A.E. Hillis, S. Chang., E. Breese and J. Heidler, The crucial role of posterior frontal regions in modality specific components of the spelling process, *Neurocase* **10** (2004), 175–187.
[20] J.H. Ferguson and F. Boller, A different form of agraphia: writing errors in patients with motor speech and movement disorders, *Brain and Language* **4** (1977), 382–389.
[21] J.A. Fiez, E.A. Raife, D.A. Balota et al., A positron emission tomography study of the short-term maintenance of verbal information, *The Journal of Neuroscience* **16** (1996), 808–822.
[22] J.R. Folk and A.C. Jones, The purpouse of lexical/sublexical interaction during spelling: further evidence from disgraphia and articulatory suppression, *Neurocase* **10** (2004), 65–69.
[23] N. Le Forestier, T. Maisonobe, L. Spelle et al., Primary lateral sclerosis: further clarification, *Journal of Neurological Sciences* **185** (2001), 95–100.
[24] F. Lucchelli and C. Papagno, Is slowly progressive anarthria a 'pure' motor-speech disorder? Evidence from writing performance, *Neurocase* **11** (2005), 234–241.
[25] A.R. Lurija, *Očerki psixofiziologii pis'ma. Izdatel'stvo Akademii pedagogičeskix nauf*, RSFSR, Moskow, 1950, Italian traslation: AR Lurija, *Neuropsicologia del linguaggio grafico*, Edizioni Messaggero, Padova, 1984.
[26] D.I. Margolin, The neuropsychology of writing and spelling: semantic, phonological, motor, and perceptual processes, *Quarterly Journal of Experimental Psychology: Human Experimental Psychology* **36A** (1984), 459–489.
[27] P.K. McGuire, D.A. Silbersweig, R.M. Murray et al., Functional anatomy of inner speech and auditory verbal imagery, *Psychological Medicine* **26** (1996), 29–40.
[28] B. Middel, H. Kuipers-Upmeijer, J. Bouma et al., Effect of intrathecal baclofen delivered by an implanted programmable pump on health related quality of life in patients with severe spasticity, *Journal of Neurology, Neurosurgery and Psychiatry* **63** (1997), 204–209.
[29] R. Moretti, P. Torre, R.M. Antonello et al., Writing errors by normal subjects, *Perceptual and Motor Skills* **97** (2003), 215–229.
[30] C. Papagno and L. Girelli, Writing through the phonological buffer: a case of progressive writing disorder, *Neuropsychologia* **43** (2005), 1277–1287.
[31] E. Paulesu, C.D. Frith and R.S.J. Frackowiak, The neural correlates of the verbal component of working memory, *Nature* **362** (1993), 342–344.
[32] W. Penfield and L. Roberts, *Speech and Brain-Mechanisms*, N.J. Princeton, Princeton University Press and London: Oxford University Press, 1959.
[33] A. Piquard, N. Le Forestier, V. Baudoin-Madec et al., Neuropsychological changes in patients with primary lateral sclerosis, *Amyotrophic Lateral Sclerosis* **7** (2006), 150–160.
[34] C.E. Pringle, A.J. Hudson, D.G. Munoz et al., Primary Lateral Sclerosis. Clinical features, neuropathology and diagnostic criteria, *Brain* **115** (1992), 495–520.
[35] S.Z. Rapcsak, S.A. Arthur and A.B. Rubens, Lexical agraphia from focal lesion of the left precentral gyrus, *Neurology* **38** (1988), 1119–1123.
[36] J.C. Raven, *Guide to using the Coloured Progressive Matrices*, London Lewis, 1965.
[37] K. Sage and A.W. Ellis, Lexical influences in graphemic buffer disorder, *Cognitive Neuropsychology* **21** (2004), 381–400.
[38] E. Service and R. Turpeinen, Working memory in spelling: evidence from backward typing, *Memory* **9** (2001), 395–421.
[39] N.O. Schiller, J.A. Greenhall, J.R. Shelton and A. Caramazza, Serial order effects in spelling errors: evidence from two dysgraphic patients, *Neurocase* **7** (2001), 1–14.
[40] M. Victor and A.H. Ropper, *Adams & Victor's Principles of Neurology*, McGraw-Hill, USA, 2005.
[41] S. Zago, B. Poletti, M. Corbo, V. Cardin and V. Silani, Dysgraphia as the presenting symptom of primary lateral sclerosis, *ALS and Other Motor Neuron Disorders* **4**(Suppl. 1) (2003), 205.
